# P12. Combination of radiotherapy and chemotherapy with dendritic cell immunotherapy in glioblastoma patients induces NK and NKT cell responses

**DOI:** 10.1186/2051-1426-2-S2-P3

**Published:** 2014-03-12

**Authors:** S Pellegatta, M Eoli, MG Bruzzone, S Cuzzubbo, E Anghileri, C Antozzi, S Frigerio, B Pollo, EA Parati, G Finocchiaro

**Affiliations:** 1 Neurological Institute C. Besta, Unit of Molecular Neuro-Oncology, Milan, Italy; 2 Neurological Institute C. Besta, Unit of Neuro-Radiology, Milan, Italy; 3 Neurological Institute C. Besta, Unit of Neuro-Immunology, Milan, Italy; 4 Neurological Institute C. Besta, Unit of Cell Therapy, Milan, Italy; 5 Neurological Institute C. Besta, Unit of Neuropathology, Milan, Italy

## Background

Two clinical studies including the treatment of first diagnosis and recurrent glioblastoma (GB) patients with dendritic cells (DCs) loaded with autologous tumour lysate (Nava et al, 2012) are currently active at Istituto Neurologico Besta, Milan.

Our first results obtained on a group of recurrent GB patients demonstrated that the response of NK cells correlates with a significantly prolonged survival (Pellegatta et al, 2013).

## Material and methods

We have examined 17 patients with primary GB receiving standard radio and chemotherapy with temozolomide (RT-TMZ) after leukapheresis and TMZ in combination with DC vaccines. The median age at surgery was 57 y (range: 23–70). Peripheral Blood Lymphocytes (PBLs) were analysed by flow cytometry to identify CD8+ T cells, NK and NKT cells before and after DC vaccines. The ratio of vaccination/baseline frequencies (V/B ratio) of all of the immunological parameters for each patient was calculated, and the median of all of the observations used as the cut off value to separate patients into the 'low' or 'high' groups.

## Results

RT-TMZ induced significant lymphopenia (<1000 lymphocytes/microl)) in 13/17 patients (76.4%). V/B ratio was correlated with the progression free survival (PFS) of each patient. Increased V/B ratio for NK cells and in particular NKT cells, but not for CD8 T lymphocytes, was significantly associated with prolonged PFS (median PFS 15 vs 8.5 mo, p=0.03; 15.0 vs 8.0 mo, p=0.002, respectively). Responder patients (PFS ≥ 12) showed increased expression of interferon (IFN)-γ immediately after the second vaccination as evaluated by real time-PCR.

No changes in the expression levels of IFN-γ were observed in the other patients.

## Conclusions

Our results encourage further investigation on the role of NK and NKT cells in anti-tumour responses and on possible interference of radio-chemotherapy on activation of CD8+ T cells.

